# Analyzing average and conditional effects with multigroup multilevel structural equation models

**DOI:** 10.3389/fpsyg.2014.00304

**Published:** 2014-04-23

**Authors:** Axel Mayer, Benjamin Nagengast, John Fletcher, Rolf Steyer

**Affiliations:** ^1^Department of Methodology and Evaluation Research, Institute of Psychology, University of JenaJena, Germany; ^2^Department of Data Analysis, Ghent UniversityGhent, Belgium; ^3^Center for Educational Science and Psychology, University of TübingenTübingen, Germany; ^4^Department of Education, University of OxfordOxford, UK

**Keywords:** multilevel analysis of covariance, average effects, multilevel structural equation modeling, conditional effects, quasi-experimental designs

## Abstract

Conventionally, multilevel analysis of covariance (ML-ANCOVA) has been the recommended approach for analyzing treatment effects in quasi-experimental multilevel designs with treatment application at the cluster-level. In this paper, we introduce the generalized ML-ANCOVA with linear effect functions that identifies average and conditional treatment effects in the presence of treatment-covariate interactions. We show how the generalized ML-ANCOVA model can be estimated with multigroup multilevel structural equation models that offer considerable advantages compared to traditional ML-ANCOVA. The proposed model takes into account measurement error in the covariates, sampling error in contextual covariates, treatment-covariate interactions, and stochastic predictors. We illustrate the implementation of ML-ANCOVA with an example from educational effectiveness research where we estimate average and conditional effects of early transition to secondary schooling on reading comprehension.

## 1. Introduction

In the social sciences, analysis of covariance (ANCOVA) is one of the most important statistical techniques. It is used to analyze effects of an independent variable on an outcome variable controlling for a vector of covariates. In this article, we focus on the application of ANCOVA-like techniques in quasi-experimental multilevel designs with treatment implementation at the level of clusters. Multilevel structural equation models (Rabe-Hesketh et al., 2004; Mehta and Neale, 2005; Marsh et al., 2009; Lüdtke et al., 2011) are used to extend traditional ANCOVA to account for the clustered structure in multilevel designs. In order to estimate causal effects in such designs, it is important to control for all relevant covariates, including covariates at the individual-level (e.g., student characteristics, intelligence, socio-economic status), contextual covariates (e.g., neighborhood-average socio-economic status, school-average achievement) and true cluster-level covariates (characteristics of the cluster, e.g., school resources, location). For extended discussions of causal effects in multilevel quasi-experiments, see Gitelman (2005), Hong and Raudenbush (2006), Sobel (2006), Hong and Raudenbush (2008), VanderWeele (2008), and Nagengast (2009).

We propose a multigroup multilevel structural equation modeling approach (MG-ML-SEM approach; see Muthén, 2002) that extends conventional multilevel ANCOVA in several ways. The particular strengths of the MG-ML-SEM approach are: (1) It allows for latent covariates at the individual-level and at the cluster-level to control for measurement error; (2) it takes into account sampling error in the aggregation of covariates to the cluster-level; (3) it naturally includes interactions between the cluster-level treatment variable and (latent) covariates at both levels and (4) all predictor variables are treated as stochastic rather than fixed predictors. The MG-ML-SEM approach is illustrated by an application from educational research in Germany (ELEMENT study, Lehmann and Lenkeit, 2008), where we estimate average and conditional effects of early transition to secondary schooling on reading achievement.

The paper is structured as follows: First, we introduce conventional multilevel ANCOVA for the analyses of quasi-experimental designs with treatment application at the cluster-level. Then, we discuss the shortcomings of this conventional approach. Next, we illustrate how the MG-ML-SEM approach can be used to overcome these shortcomings for estimating average and conditional treatment effects and compare several models of the MG-ML-SEM approach. In the discussion, we point out the assumptions required to estimate *causal* effects and discuss further research directions.

## 2. Multilevel designs with treatment application at the cluster-level

Multilevel designs with two or more treatment groups are typically differentiated along the dimensions of (a) the level of treatment application (at the individual- or the cluster-level) and (b) the treatment assignment process (randomized or non-randomized assignment) (e.g., Plewis and Hurry, 1998; Ukoumunne et al., 1999). In the remainder, we will focus on a class of multilevel designs that are of particular importance for educational research: Designs in which whole clusters are treated and there are systematic differences between clusters. Such quasi-experimental designs are particularly relevant when randomized assignment to conditions is not possible, for example when the effects of differential learning environments or school types are studied.

As in all quasi-experimental designs, observed group differences do not reflect unbiased treatment effects in multilevel designs with non-random assignment at the cluster-level, because confounding variables can bias the estimates of treatment effects. Therefore it is crucial to measure and statistically control such confounders (e.g., Rosenbaum, 2002; Shadish et al., 2002). In the remainder, we call a potential confounder a covariate.

Conventionally, multilevel ANCOVA has been the recommended approach for analyzing quasi-experimental multilevel designs with treatment application at the cluster-level (e.g., Plewis and Hurry, 1998; Seltzer, 2004). Multilevel ANCOVA for a design with two conditions includes an indicator for the treatment condition *X* and controls for *k* covariates *Z*_1_,…,*Z*_*k*_. A typical implementation of an hierarchical two-level ANCOVA, for example given in Raudenbush and Bryk (2002), p. 26, Equation (2.16) is as follows:

(1) $$Y=\gamma_{00}+\gamma_{10}\cdot X+\gamma_{20}\cdot Z_{1}+\ldots +\gamma_{k0}\cdot Z_{k}+\underset{\varepsilon }{\underset{︸}{u+r}}.$$

If this model is appropriately specified, i.e., if all covariates are included and there are no unmodeled non-linear effects or treatment covariate interactions, γ_10_ is an estimate of the average treatment effect *AVE* (e.g., Aiken and West, 1996). In order to obtain correct standard errors, the model should be implemented as a hierarchical linear model to take the clustered structure of the design into account (e.g., Murray, 1998, 2001; Seltzer, 2004), i.e., the residual variable ε can be decomposed into a cluster-level component *u*, that reflects the residual variation between classrooms and an individual level-component *r*, that reflects variability of individual students around the values predicted by the covariates and their cluster. The model can be easily extended by including cluster-level covariates *W* to control for selection effects that operate at the cluster-level.

## 3. Limitations of conventional multilevel ANCOVA

The conventional multilevel ANCOVA presented in Equation (1), however, has several drawbacks that limit its potential for the analyses of quasi-experimental multilevel designs with treatment application at the cluster-level.

### 3.1. Measurement error

Conventional multilevel ANCOVA implemented in the hierarchical linear model does not take measurement error into account, leading to potentially biased estimates of treatment effects (Cook et al., 2009; Culpepper and Aguinis, 2011; Lockwood and McCaffrey, 2014). It is well known that unreliably measured covariates can yield biased results in regression analysis (Degracie and Fuller, 1972; Carroll et al., 2010). This problem is exacerbated in multilevel designs in educational and psychological research, where many covariates can only be measured unreliably and latent covariates can appear at several levels. The emergence of multilevel structural equation models (Rabe-Hesketh et al., 2004; Mehta and Neale, 2005; Marsh et al., 2009; Lüdtke et al., 2011) allows for controlling measurement error in covariates (and outcomes) by including multiple indicators both at the individual- and cluster-level. Multilevel structural equation models also allow for analyzing the factorial structure of latent variables and tests of measurement invariance across levels (Mehta and Neale, 2005; Jak et al., 2013)—two assumptions of conventional multilevel ANCOVA that are not routinely tested (see also Schweig, 2013).

### 3.2. Contextual effects and sampling error

In quasi-experimental designs with treatment application at the cluster-level, *contextual covariates* are especially important. Contextual covariates (sometimes also referred to as compositional variables, e.g., Harker and Tymms, 2004; Hutchison, 2007) reflect the composition of cluster-level units, for example the average achievement level in a classroom or the average socio-economic status in a neighborhood. Contextual covariates are particularly important covariates because it is very likely that they are associated with selection processes at the cluster-level.

Formally, contextual covariates are conditional expectations of a unit-level covariate *Z* given the cluster variable *C*. Every unit-level covariate *Z* can be decomposed into a contextual covariate or *between-cluster component Z*_*b*_ and a residual or *within-cluster component Z*_*w*_:

(2) $$\begin{array}{l} Z=E\left(Z\;|\;C\right)+Z_{w}\\ \;=Z_{b}+Z_{w}, \end{array}$$

where *Z*_*w*_ = *Z* − *E*(*Z* | *C*) (Lüdtke et al., 2008; Snijders and Bosker, 2012). This decomposition shows that a contextual covariate *Z*_*b*_ is the regression of the individual-level covariate *Z* on the cluster-variable *C*. As such, all properties of a regression residual hold for the within-cluster component *Z*_*w*_. Notably, its expected value is equal to zero and it is regressively independent of the cluster variable *C* as well as of all functions of the cluster-variable such as other covariates at the cluster-level.

The notion of “contextual effects”—the differential effects of the within-cluster and between-cluster components on the outcome variable—has long been discussed (Raudenbush and Willms, 1995). It is important to take account of such differential effects when controlling for cluster-level differences in multilevel models and it is critical to the unbiased estimation of treatment effects when selection into different treatments occurs at the cluster level. The conventional multilevel ANCOVA of Equation (1) does not make this distinction. The coefficient γ_*k*0_ represents the total effect of the covariate *Z*_*k*_ that is a blend of the effects of *Z*_*kb*_ and *Z*_*kw*_. The value of γ_*k*0_ depends on the intraclass-correlation-coefficient of the covariate *Z*_*k*_ (Raudenbush and Bryk, 2002; Snijders and Bosker, 2012). If only *Z*_*k*_ is included as covariate in an ANCOVA, but selection into treatment conditions varies as a function of *Z*_*kb*_, the adjusted effects will be biased (Nagengast, 2009).

In applications another difficulty arises: The values of contextual covariates *Z*_*b*_ are often estimated by the manifest sample mean in each cluster, assuming perfect reliability of cluster means. This assumption is unrealistic if there is only a sample of individuals taken from each cluster or if the individual-level ratings measure a cluster-level construct such as classroom climate (Marsh et al., 2012). Recently, new approaches have been developed that take the unreliability of cluster means into account (Croon and van Veldhoven, 2007; Lüdtke et al., 2008; Shin and Raudenbush, 2010; Grilli and Rampichini, 2011). Marsh et al. (2009) and Lüdtke et al., (2011) introduced *doubly-latent models* that further extend these approaches by allowing for latent aggregation of latent variables measured by multiple indicators. They also demonstrated that the contextual effect, i.e., the effect of the contextual variable *Z*_*b*_ after controlling for the effects of the unit-level covariate *Z*, will be biased when sampling error is not controlled. This bias, that is similar to the bias due to measurement error, will also affect the estimation of treatment effects when a contextual covariate is included in the model.

### 3.3. Treatment-covariate interactions

The literature on ANCOVA for multilevel designs with treatment application at the cluster-level has been surprisingly sparse on the issue of including interactions between the treatment and various covariates at the unit- and the cluster-level (see, Plewis and Hurry, 1998; Pituch, 2001; Seltzer, 2004, for notable exceptions). Such interactions indicate that the effect of the treatment is not constant across all units and clusters, but depends on the values of individual- and cluster-level covariates. Interactions contain important information about the differential effectiveness of the treatment for subgroups of units or clusters. In the presence of interactions, researchers may consider conditional treatment effects (i.e., treatment effects given particular values of the covariate(s) and/or the treatment), or the average treatment effect (i.e., the treatment effect obtained by averaging the conditional treatment effects over the unconditional distribution of covariates). In models ignoring important interactions, the regression coefficient for the treatment effect is an aggregate of the conditional treatment effects, but is *not* equal to the average treatment effect (see, Rogosa, 1980, for a detailed explanation in the context of single-level models). Using an example with a single covariate, Rogosa (1980) showed that the treatment effect obtained from traditional ANCOVA (misspecified by ignoring the interaction) is an estimate of the (*Z* = *z*_*ca*_)-conditional treatment effect, where *z*_*ca*_ denotes the center of accuracy. The center of accuracy is the point, where the conditional variance of the effect function is minimal and it is not necessarily equal to the average of *Z*.

### 3.4. Stochastic regressors

The conventional hierarchical linear model explicitly assumes that the predictors within a sample are fixed quantities that do not vary from sample to sample (see e.g., Pinheiro and Bates, 2000; Raudenbush and Bryk, 2002; Snijders and Bosker, 2012). Hence, all inferences are conditional on the values of the set of observed covariates in the sample (Senn et al., 2007). While this assumption simplifies the implementation of the statistical estimation procedure, the case has been made that it is not appropriate for the analysis of quasi-experimental designs and observational studies (Crager, 1987; Chen, 2006; Nagengast, 2006; Kröhne, 2009) when unconditional inferences to the true distribution (and not to the sample distribution) of covariates are desired. In these designs, it is unlikely that the distribution of covariates in a sample would be identical in a replication of the study and treating the covariates as fixed predictors is not appropriate. Kröhne (2009) showed analytically and in simulation studies that standard errors of average effects obtained from the conventional general linear model assuming fixed predictors will be biased in the presence of treatment-covariate interactions if the covariate is, in fact, a stochastic predictor (see also Sampson, 1974). While the problem of stochastic predictors and unconditional inference has received some attention in experimental design (Gatsonis and Sampson, 1989), correlation analysis (Shieh, 2006) and power analysis (Steiger and Fouladi, 1992) for the general linear model, the topic has not been studied widely for hierarchical linear models.

## 4. Generalized ANCOVA

Before we discuss the MG-ML-SEM framework in more detail, we first introduce *generalized ANCOVA* (Steyer and Partchev, 2008) that was developed to overcome some of the issues of conventional ANCOVA in single-level designs. For a binary treatment indicator *X* and a multivariate covariate *Z* = (*Z*_1_,…,*Z_k_*), the regression of *Y* on *X* and *Z* can always be written as

(3) $$E\left(Y\;|\;X,Z\right)=g_{0}(Z)+g_{1}(Z)\cdot X.$$

In this representation of the regression, the *intercept function g*_0_(*Z*) describes the conditional regressive dependency of the outcome *Y* and the covariates in the control group (i.e., for *X* = 0). The values of the *effect function g*_1_(*Z*) are the conditional treatment effects given particular values *z* of the covariate *Z*.

In order to estimate treatment effects, one has to choose a parameterization for both *g*_0_(*Z*) and *g*_1_(*Z*). Often, linear parameterizations are chosen for the intercept and the effect functions (e.g., Aiken and West, 1996) although other parameterizations, e.g., non-linear functions are also possible. Using only a single covariate *Z* and assuming linear functions for *g*_0_(*Z*) and *g*_1_(*Z*) yields

(4) $$E\left(Y\;|\;X,Z\right)=\left(\gamma_{00}+\gamma_{10}\cdot Z\right)+\left(\gamma_{01}+\gamma_{11}\cdot Z\right)\cdot X.$$

Equation (4) extends conventional ANCOVA by including the interaction term γ_11_. If there is an interaction effect, i.e., if γ_11_ ≠ 0, treatment effects are not constant, but vary as a linear function of the covariate *Z*.

Based on Equation (4), one can also obtain the average effect of the treatment (*AVE*) by taking the expectation of the effect function *g*_1_(*Z*):

(5) $$\begin{array}{l} AVE=E\left(\gamma_{01}+\gamma_{11}\cdot Z\right)\\ \;=\gamma_{01}+\gamma_{11}\cdot E(Z). \end{array}$$

Hence, in the presence of interaction effects, the *AVE* is no longer represented by a single parameter as in conventional ANCOVA, but is identified by a non-linear function of regression coefficients and the expected value of the covariate. If the expected value of the covariate *E*(*Z*) were included as a model parameter, e.g., in a multigroup structural equation model (Kröhne, 2009; Nagengast, 2009), the estimation of the *AVE* using Equation (5) could take into account the uncertainty associated with the covariate means that is introduced when covariates are stochastic predictors (Sampson, 1974; Gatsonis and Sampson, 1989; Steiger and Fouladi, 1992; Chen, 2006; Shieh, 2006). In contrast, the more common approach of mean centering the covariate(s) to obtain average effects (Aiken and West, 1996) ignores this uncertainty. Based on Equation (4), one can also obtain conditional treatment effects *CTE* = *g*_1_(*z*) given particular values *z* of *Z*:

(6) $$g_{1}(z)=\gamma_{01}+\gamma_{11}\cdot z,$$

which are also identified by non-linear functions of regression coefficients and a value *z* of *Z*. While generalized ANCOVA and its implementation into multigroup SEM solves some of the problems of ANCOVA without interaction, further steps are necessary in order to account for contextual covariates and measurement error in quasi-experimental multilevel designs.

## 5. MG-ML-SEM implementation of generalized ML-ANCOVA

In the remainder of this paper, we introduce the MG-ML-SEM implementation of generalized ML-ANCOVA as an alternative to the conventional implementation of ML-ANCOVA models in hierarchical linear models. The MG-ML-SEM approach naturally overcomes the limitations of the conventional approach mentioned above: (1) It controls for measurement error by including measurement models for multiple indicators of covariates and outcomes. (2) It easily allows the inclusion of contextual effects with the appropriate controls for sampling error by the latent aggregation approach. (3) It includes treatment-covariate interactions as a default as it is based on a multiple-group multilevel SEM model. (4) All predictors are treated as stochastic rather than fixed quantities. In addition, the MG-ML-SEM approach allows for group-specific variances of the dependent variable *Y* given covariates *Z*.

### 5.1. MG-ML-SEM

The implementation of generalized ML-ANCOVA is based on the multilevel structural equation model of Muthén (1989, 1994) and its extension to multigroup multilevel structural equation models (Muthén et al., 1997). Note that it is also possible to present and implement the model in the GLLAMM-framework (Rabe-Hesketh et al., 2004; Skrondal and Rabe-Hesketh, 2004). Rabe-Hesketh et al. (2012) provide a discussion of the advantages of different frameworks for multilevel structural equation modeling.

The MG-ML-SEM decomposes the vector of manifest variables (**Y**, **Z**, **W_b_**) into the cluster-level variables (Y^*^_b_, Z^*^_b_, **W_b_**) and individual-specific variables Y^*^_w_, Z^*^_w_, **0**) (see Equation 2). The elements of these vectors can be modeled in two ways—either using group-mean centering of unit-level variables and the corresponding group means as additional predictors (Kreft et al., 1995; Raudenbush and Bryk, 2002; Enders and Tofighi, 2007) or using a full-information latent aggregation approach (Lüdtke et al., 2008). Similar to the correction of the between-cluster variance of the outcome variable in conventional multilevel models (e.g., Snijders and Bosker, 2012), the latter approach accounts for the fact that the observed between-cluster variances and covariances of the predictors are biased estimators of the true between-cluster variances and covariances. It corrects the effects of the between-cluster covariance matrix for the effects of the within-cluster variances and covariances. Throughout this article, we will denote the latent aggregation approach with a superscript of an asterisk^1^. This decomposition is given by:

(7) $$\begin{array}{l} (\begin{array}{c} \begin{array}{c} Y \end{array}\\ Z\\ W_{b} \end{array})=(\begin{array}{c} \begin{array}{c} Y_{b}^{*} \end{array}\\ Z_{b}^{*}\\ W_{b} \end{array})+(\begin{array}{c} \begin{array}{c} Y_{w}^{*} \end{array}\\ Z_{w}^{*}\\ 0 \end{array}), \end{array}$$

where **Y** is the vector of manifest indicators of latent variables, **Z** is the vector of manifest covariates measured at the unit-level, and **W_b_** is the vector of true cluster-level covariates. The vector (Y^*^_b_, Z^*^_b_, **W_b_**) contains the latent between-cluster components of the variables and the vector Y^*^_w_, Z^*^_w_, **0**) contains the latent within-cluster components of the corresponding variables on the unit-level^2^.

The MG-ML-SEM consists of (1) the group-specific within-cluster measurement model, (2) the group-specific between-cluster measurement model, (3) the group-specific within-cluster structural model, and (4) the group-specific between-cluster structural model:

$$\begin{array}{ll} (\begin{array}{c} \begin{array}{c} Y_{w}^{*} \end{array}\\ Z_{w}^{*} \end{array})=\Lambda_{wx}\eta_{w}^{*}+\varepsilon_{w} & \begin{array}{l} \text{within-cluster measurement}\\ \text{model for }X=x \end{array}\\ (\begin{array}{c} Y_{b}^{*}\\ Z_{b}^{*}\\ W_{b} \end{array})=\nu_{x}+\Lambda_{bx}\eta_{b}^{*}+\varepsilon_{b} & \begin{array}{l} \text{between-cluster measurement}\\ \text{model for }X=x \end{array}\\ \;\eta_{w}^{*}=A_{x}\eta_{w}^{*}+\zeta_{w} & \begin{array}{l} \text{within-cluster structural}\\ \text{model for }X=x \end{array}\\ \;\eta_{b}^{*}=\beta_{x0}+B_{x}\eta_{b}^{*}+\zeta_{b} & \begin{array}{l} \text{between-cluster structural}\\ \text{model for }X=x \end{array} \end{array}$$

See Muthén (2004) for details on the implementation of the MG-ML-SEM as a sampling model.

Considering only one individual-specific covariate *Z* = Z^*^_b_ + Z^*^_w_, the structural model of the MG-ML-SEM is used to estimate the group-specific regressions *E*^*X* = *x*^(*Y* | Z^*^_b_, Z^*^_w_):

(8) $$\begin{array}{l} \begin{array}{l} E^{X=x}\left(Y\;|\;Z_{b}^{*},Z_{w}^{*}\right)=E^{X=x}\left(Y_{b}^{*}\;|\;Z_{b}^{*},Z_{w}^{*}\right)\\ \;+E^{X=x}\left(Y_{w}^{*}\;|\;Z_{b}^{*},Z_{w}^{*}\right)\\ \;=E^{X=x}\left(Y_{b}^{*}\;|\;Z_{b}^{*}\right)+E^{X=x}\left(Y_{w}^{*}\;|\;Z_{w}^{*}\right)\\ \;=\beta_{x0}+\beta_{x1}\cdot Z_{b}^{*}+\alpha_{x1}\cdot Z_{w}^{*}, \end{array} \end{array}$$

where Y^*^_b_, Z^*^_b_ are (possibly latent) variables in η^*^_b_, Y^*^_w_, Z^*^_w_ are (possibly latent) variables in η^*^_w_, α_*x*1_ is a within-cluster regression coefficient in **A**_*x*_, β_*x*0_ is a between-cluster intercept in **β_*x*0_**, and β_*x*1_ is a between-cluster regression coefficient in **B**_*x*_.

### 5.2. Generalized ML-ANCOVA

The generalized ML-ANCOVA combines aspects of conventional ML-ANCOVA (by considering the nested structure and within- and between-cluster components of variables) and aspects of generalized single-level ANCOVA (by considering interactions between the treatment variable and covariates).

The generalized ML-ANCOVA for a single covariate *Z* = Z^*^_b_ + Z^*^_w_ and a dichotomous treatment variable *X* with values 0 and 1 is given by:

(9) $$E\left(Y\;|\;X,Z_{b}^{*},Z_{w}^{*}\right)=g_{0}\text{​}\left(Z_{b}^{*},Z_{w}^{*}\right)+g_{1}\text{​}\left(Z_{b}^{*},Z_{w}^{*}\right)\cdot X.$$

Since we want to analyze average and conditional treatment effects, our main interest lies in the effect function *g*_1_ (Z^*^_b_, Z^*^_w_). In the multi-group setting, the effect function can be computed as the difference between the two group-specific regressions *E*^*X* = *x*^(*Y* | Z^*^_b_, Z^*^_w_):

(10) $$g_{1}\text{​}\left(Z_{b}^{*},Z_{w}^{*}\right)=E^{X=1}\left(Y\;|\;Z_{b}^{*},Z_{w}^{*}\right)-E^{X=0}\left(Y\;|\;Z_{b}^{*},Z_{w}^{*}\right).$$

Inserting Equation (8) into (10) yields the effect function of generalized ML-ANCOVA based on parameters of the MG-ML-SEM:

(11) $$\begin{array}{l} g_{1}\text{​}\left(Z_{b}^{*},Z_{w}^{*}\right)=E^{X=1}\left(Y\;|\;Z_{b}^{*},Z_{w}^{*}\right)-E^{X=0}\left(Y\;|\;Z_{b}^{*},Z_{w}^{*}\right)\\ \;=\left(\beta_{10}-\beta_{00}\right)+\left(\beta_{11}-\beta_{01}\right)\cdot Z_{b}^{*}+\left(\alpha_{11}-\alpha_{01}\right)\cdot Z_{w}^{*}\\ \;=\gamma_{10}+\gamma_{11}\cdot Z_{b}^{*}+\gamma_{12}\cdot Z_{w}^{*}. \end{array}$$

This effect function can be used to compute average and conditional effects. In order to compute the average treatment effect, we first need to compute the unconditional expectations of covariates. The MG-ML-SEM only contains parameters for the conditional expectations of covariates given treatment group *x*, but the unconditional expectation can be computed by:

(12) $$\begin{array}{l} E(Z)=E\left[E(Z\;|\;C)\right]=E(Z_{b})=E\left[E(Z\;|\;X)\right]\\ \;=\mu_{01}\cdot P\left(X=0\right)+\mu_{11}\cdot P\left(X=1\right), \end{array}$$

where μ_01_, μ_11_ are between-cluster “intercepts” of exogenous covariates in **β_*x*0_**, i.e., μ_01_, μ_11_ are the group-specific true means μ_01_= *E*(*Z* | *X* = 0) and μ_11_= *E*(*Z* | *X* = 1). Then, the average effect is *AVE* = *E*[*g*_1_(Z^*^_b_, Z^*^_w_)] = γ_10_ + γ_11_ · *E*(*Z*).

Generalized ML-ANCOVA can easily be extended to *j* + 1 treatment groups and to include more covariates at the within- and at the between-cluster level, as well as pure cluster level covariates:

(13) $$\begin{array}{l} E\left(Y\;|\;X,Z_{b}^{*},Z_{w}^{*},W\right)=g_{0}\text{​}\left(Z_{b}^{*},Z_{w}^{*},W\right)+g_{1}\left(Z_{b}^{*},Z_{w}^{*},W\right)\cdot \\ \;I_{X=1}+\ldots +g_{j}\text{​}\left(Z_{b}^{*},Z_{w}^{*},W\right)\cdot I_{X=j}, \end{array}$$

where *I*_*X* = *j*_ is an indicator for treatment condition *j*, and Z^*^_b_, Z^*^_w_, *W* are multivariate random variables. Based on Equation (13), we can consider average treatment effects *E*[*g*_*j*_(Z^*^_b_, Z^*^_w_, *W*)] and conditional treatment effects *E*[*g*_*j*_(Z^*^_b_, Z^*^_w_, *W*) | *f*(Z^*^_b_, Z^*^_w_, *W*, *X*)] given any function *f*(Z^*^_b_, Z^*^_w_, *W*, *X*).

Next, we illustrate the MG-ML-SEM approach to generalized ML-ANCOVA with a model from educational effectiveness research. Using a single dataset, we present the increasingly complex MG-ML-SEM models that show the features and the versatility of the approach.

## 6. Illustrative example

### 6.1. Participants and procedure

To illustrate the MG-ML-SEM, we use data from the ELEMENT study in Berlin, Germany (Lehmann and Lenkeit, 2008). ELEMENT is a three wave longitudinal study aimed at examining effects of early transition to secondary school (after 4th grade) on students' reading and mathematics proficiency. From 2002/2003 to 2004/2005, a total of *N* = 4926 students were measured in 4th grade, 5th grade, and 6th grade with several ability tests. *N* = 3169 students attended elementary school until the end of 6th grade, whereas *N* = 1757 decided to make the transition to secondary school after 4th grade.

In this article, we do not present a comprehensive analysis of the ELEMENT study. Instead, the primary goal of our paper is to illustrate the MG-ML-SEM approach as a means to estimate conditional and average effects in educational research. For didactic purposes, we restrict ourselves to reading comprehension as outcome, to a limited set of covariates (prior reading achievement and interest in reading), to two occasions of measurement (4th grade and 6th grade), and to only one of the five data sets with imputed missing values provided by the Research Data Centre at the Institute for Educational Quality Improvement. A complete causal analysis would most likely require the inclusion of more covariates and/or propensity scores (see causal inference section in the discussion for details), and careful consideration of the sampling design and the missing data structure (e.g., Baumert et al., 2009; Lehmann, 2010; Becker et al., 2014).

### 6.2. Measures

#### 6.2.1. Reading comprehension

The reading test used in the ELEMENT study was based on the theoretical framework of the IEA and OECD reading assessments. It contained items from PIRLS (Mullis et al., 2006) and from the LAU Study (Lehmann et al., 1997, 1999). Students' reading abilities were obtained as weighted likelihood estimates based on an IRT model. In order to obtain comparable scores across time, an anchor items design was used. In our analyses, we used test scores from 4th grade as covariate *Z* in order to control for pre-existing differences in reading, and we used test scores from 6th grade as outcome variable *Y*.

#### 6.2.2. Interest in reading

The scale “interest in reading” consisted of five items. For our analyses including a latent covariate η, we used the following three positively worded items as indicators of a common latent variable:

**Table:** 

*V*_1_ (Item Asf0902):	I like talking to others about books.
*V*_2_ (Item Asf0903):	I am pleased with a book received as a gift.
*V*_3_ (Item Asf0905):	I like reading.

The response format was a four-point Likert scale with categories “1 = strongly agree,” “2 = agree,” “3 = disagree,” and “4 = strongly disagree.” The three items were recoded so that higher values represent higher interest in reading. For the analyses including only manifest variables, we computed a scale *V* for “interest in reading” by taking the mean of the three recoded items for each student.

#### 6.2.3. Other variables

Our treatment variable *X* is type of school at 6th grade with values *X* = 0 (elementary school) and *X* = 1 (secondary school). We used class ID in 6th grade as cluster variable *C*. For some of the analyses ignoring latent aggregation, we created between-cluster components of all covariates by computing the empirical class means. We also created the within-cluster components by computing the difference between the corresponding variable and their empirical class means. This decomposition was done for reading comprehension at 4th grade *Z*, each of the three indicators of “interest in reading” *V*_1_, *V*_2_, *V*_3_, and the scale for “interest in reading” *V* = (*V*_1_ + *V*_2_ + *V*_3_)/3.

## 7. Models

We analyzed the data with six multigroup multilevel models in order to illustrate the different effect estimates and highlight the strengths of the MG-ML-SEM approach. In the simplest model M1, we did not control for any covariates; in M2, we controlled for manifest covariates without distinguishing within and between components (as in a traditional multilevel ANCOVA model); in M3, we controlled for within and between components of manifest covariates using manifest aggregation; in M4, we controlled for within and between components of manifest covariates using latent aggregation; in M5, we controlled for within and between components of a latent and a manifest covariate using manifest aggregation; and finally in M6, we specified the full doubly latent model controlling for within and between components of a latent and a manifest covariate using latent aggregation. See Table 1 for an overview of models and their characteristics. We chose to present all six models in order to show differences in point estimates and standard errors between models in the context of our illustrative example. These analyses show considerable differences between models and demonstrate the need for a careful examination of treatment effects while meeting the requirements of complex multilevel designs.

**Table 1:** **Overview of six multigroup multilevel structural equation models and their characteristics**.

	**M1**	**M2**	**M3**	**M4**	**M5**	**M6**
Covariates		✓	✓	✓	✓	✓
Contextual covariates			✓	✓	✓	✓
Latent covariates					✓	✓
Latent aggregation				✓		✓

### 7.1. M1: MG-ML-SEM without covariates

The first MG-ML-SEM is a way of estimating the (unadjusted) means of reading comprehension from 6th grade *Y* in elementary school (*X* = 0) and secondary school (*X* = 1). The parameters in this model are the group-specific within and between variances of *Y*, and the group-specific means of *Y*. The within and between structural model for both treatment groups (*X* = 0 and *X* = 1) are given by:

$$\begin{array}{l} Y_{w}^{*}=0+\zeta_{w}\text{ within structural model }X=0\\ Y_{w}^{*}=0+\zeta_{w}\text{ within structural model }X=1\\ Y_{b}^{*}=\beta_{00}+\zeta_{b}\text{ between structural model }X=0\\ Y_{b}^{*}=\beta_{10}+\zeta_{b}\text{ between structural model }X=1 \end{array}$$

where Y^*^_w_ is the within-cluster component of *Y* and Y^*^_b_ is the between-cluster component of *Y*. The asterisk indicates that Y^*^_w_ and Y^*^_b_ are latent variables obtained by latent aggregation. In this model without covariates, the structural intercepts β_00_ and β_10_ are the group-specific means of *Y*, ζ_*w*_ is a residual at the within level, and ζ_*b*_ is a residual at the between level.

Based on the parameters of this model, we can compute the effect function (see Equation 11), which is identical with the average treatment effect in this model, because there are no covariates:

$$AVE=\beta_{10}-\beta_{00}$$

Obviously, in the ELEMENT study as in other observational studies, the *AVE* obtained from M1 does not reflect an unbiased estimate of the average causal effect of school type on reading comprehension from 6th grade. It is only in randomized controlled trials, that we can estimate the causal effect without bias using a model such as M1, i.e., without controlling for covariates.

### 7.2. M2: MG-ML-SEM manifest covariates/without contextual covariates

In the second MG-ML-SEM, we implemented the conventional multilevel ANCOVA by adding two covariates to the model, namely reading comprehension from 4th grade *Z* and the scale score of interest in reading *V*, and consider the group-specific regressions *E*^*X* = *x*^(*Y* | *Z*, *V*) in the following MG-ML-SEM:

$$\begin{array}{l} \;Y_{w}^{*}=0+\alpha_{01}\cdot Z_{w}+\alpha_{02}\cdot V_{w}+\zeta_{w}\\ \text{ within structural model }X=0\\ \;Y_{w}^{*}=0+\alpha_{11}\cdot Z_{w}+\alpha_{12}\cdot V_{w}+\zeta_{w}\\ \text{ within structural model }X=1\\ (\begin{array}{c} Y_{b}^{*}\\ Z_{b}\\ V_{b} \end{array})=(\begin{array}{c} \begin{array}{c} \beta_{00} \end{array}\\ \mu_{01}\\ \mu_{02} \end{array})+(\begin{array}{c} \begin{array}{c} \beta_{01} \end{array}\\ 0\\ 0 \end{array})Z_{b}+(\begin{array}{c} \begin{array}{c} \beta_{02} \end{array}\\ 0\\ 0 \end{array})V_{b}+(\begin{array}{c} \begin{array}{c} \zeta_{1b} \end{array}\\ \zeta_{2b}\\ \zeta_{3b} \end{array})\\ \text{ between structural model }X=0\\ (\begin{array}{c} \begin{array}{c} Y_{b}^{*} \end{array}\\ Z_{b}\\ V_{b} \end{array})=(\begin{array}{c} \begin{array}{c} \beta_{10} \end{array}\\ \mu_{11}\\ \mu_{12} \end{array})+(\begin{array}{c} \begin{array}{c} \begin{array}{c} \beta_{11} \end{array} \end{array}\\ 0\\ 0 \end{array})Z_{b}+(\begin{array}{c} \begin{array}{c} \beta_{12} \end{array}\\ 0\\ 0 \end{array})V_{b}+(\begin{array}{c} \begin{array}{c} \zeta_{1b} \end{array}\\ \zeta_{2b}\\ \zeta_{3b} \end{array})\\ \text{ between structural model }X=1 \end{array}$$

In this model we demonstrate the consequences of disregarding the decomposition of (*V*, *Z*) into within and between components. This decomposition was ignored by constraining the corresponding regression coefficients to be equal across levels, i.e., α_01_ = β_01_, α_02_ = β_02_, α_11_ = β_11_, α_12_ = β_12_.

Based on the parameters of this model, we can compute the effect function *g*_1_(*Z*, *V*) and the average effect *AVE* as follows (see Equations 11, 12 for details):

$$\begin{array}{l} g_{1}(Z,V)=E^{X=1}(Y\;|\;Z,V)-E^{X=0}(Y\;|\;Z,V)\\ \;=(\beta_{10}-\beta_{00})+(\beta_{11}-\beta_{01})\cdot Z+(\beta_{12}-\beta_{02})\cdot V\\ \;=\gamma_{10}+\gamma_{11}\cdot Z+\gamma_{12}\cdot V\\ \;AVE=(\beta_{10}-\beta_{00})+(\beta_{11}-\beta_{01})\cdot E(Z)+(\beta_{12}-\beta_{02})\cdot E(V). \end{array}$$

The average treatment effect of school type on reading comprehension from 6th grade obtained from this model can be causally interpreted only under strong assumptions (see causal inference section in the discussion). If there are other confounders not included in the model or if there are contextual effects, the *AVE* from M2 must not be causally interpreted.

### 7.3. M3: MG-ML-SEM manifest covariates/manifest aggregation

In the third MG-ML-SEM, we add contextual covariates to the model, i.e., we decompose the manifest covariates (*Z*, *V*) into between-cluster components (*Z_b_*, *V_b_*) and within-cluster components (*Z_w_*, *V_w_*). For this decomposition, we follow the group-mean centering approach assuming perfect reliability of manifest cluster means of covariates. In the terminology of Marsh et al. (2009), M3 is called a “Doubly-Manifest Model.” We computed the values of the between-cluster variables (*Z_b_*, *V_b_*) and the within-cluster variables (*Z_w_*, *V_w_*) before fitting the model. We consider the group-specific regressions *E*^*X* = *x*^(*Y* | *Z_b_*, *V_b_*, *Z_w_*, *V_w_*) in the following MG-ML-SEM:

$$\begin{array}{l} \;Y_{w}^{*}=0+\alpha_{01}\cdot Z_{w}+\alpha_{02}\cdot V_{w}+\zeta_{w}\\ \text{ within structural model }X=0\\ \;Y_{w}^{*}=0+\alpha_{11}\cdot Z_{w}+\alpha_{12}\cdot V_{w}+\zeta_{w}\\ \text{ within structural model }X=1\\ (\begin{array}{c} Y_{b}^{*}\\ Z_{b}\\ V_{b} \end{array})=(\begin{array}{c} \begin{array}{c} \beta_{00} \end{array}\\ \mu_{01}\\ \mu_{02} \end{array})+(\begin{array}{c} \begin{array}{c} \beta_{01} \end{array}\\ 0\\ 0 \end{array})Z_{b}+(\begin{array}{c} \begin{array}{c} \beta_{02} \end{array}\\ 0\\ 0 \end{array})V_{b}+(\begin{array}{c} \begin{array}{c} \zeta_{1b} \end{array}\\ \zeta_{2b}\\ \zeta_{3b} \end{array})\\ \text{ between structural model }X=0\\ (\begin{array}{c} \begin{array}{c} Y_{b}^{*} \end{array}\\ Z_{b}\\ V_{b} \end{array})=(\begin{array}{c} \begin{array}{c} \begin{array}{c} \beta_{10} \end{array} \end{array}\\ \mu_{11}\\ \mu_{12} \end{array})+(\begin{array}{c} \begin{array}{c} \beta_{11} \end{array}\\ 0\\ 0 \end{array})Z_{b}+(\begin{array}{c} \begin{array}{c} \begin{array}{c} \beta_{12} \end{array} \end{array}\\ 0\\ 0 \end{array})V_{b}+(\begin{array}{c} \begin{array}{c} \begin{array}{c} \zeta_{1b} \end{array} \end{array}\\ \zeta_{2b}\\ \zeta_{3b} \end{array})\\ \text{ between structural model }X=1 \end{array}$$

Again, based on the parameters of M3, we can compute the effect function *g*_1_(*Z_b_*, *V_b_*, *Z_w_*, *V_w_*) and the average treatment effect as follows (see Equations 11, 12):

$$\begin{array}{l} g_{1}(Z_{b},V_{b},Z_{w},V_{w})=(\beta_{10}-\beta_{00})+(\beta_{11}-\beta_{01})\cdot Z_{b}\\ \;+\;(\beta_{12}-\beta_{02})\cdot V_{b}+(\alpha_{11}-\alpha_{01})\cdot Z_{w}\\ \;+\;(\alpha_{12}-\alpha_{02})\cdot V_{w}\\ \;=\gamma_{10}+\gamma_{11}\cdot Z_{b}+\gamma_{12}\cdot V_{b}+\gamma_{13}\cdot Z_{w}\\ \;+\;\gamma_{14}\cdot V_{w}\\ \;AVE=(\beta_{10}-\beta_{00})+(\beta_{11}-\beta_{01})\cdot E(Z)\\ \;+\;(\beta_{12}-\beta_{02})\cdot E(V) \end{array}$$

The treatment effects in this model depend on the values of the within-cluster components and the between-cluster components of covariates. Note that the equation for computing the *AVE* simplifies considerably, because the expectation of the within-cluster components of the covariates are zero, i.e., *E*(*Z_w_*) = *E*(*V_w_*) = 0 (see section 3.2). The unconditional expectations of covariates are computed as shown in Equation (12). M3 is less restrictive compared to M2. It requires causal unbiasedness of the regression *E*(*Y* | *X*, *Z_b_*, *V_b_*, *Z_w_*, *V_w_*), which means that there must not be any omitted confounders.

### 7.4. M4: MG-ML-SEM manifest covariates/latent aggregation

Our fourth MG-ML-SEM is very similar to M3, with the only exception that contextual covariates are treated as latent variables using the full-information latent aggregation approach (Lüdtke et al., 2008) as indicated by the superscript of an asterisk (Z^*^_w_, Z_b_^*^, V_w_^*^, V_b_^*^) in the model equations for the fourth MG-ML-SEM. This model was first presented in Nagengast (2009):

$$\begin{array}{l} \;Y_{w}^{*}=0+\alpha_{01}\cdot Z_{w}^{*}+\alpha_{02}\cdot V_{w}^{*}+\zeta_{w}\\ \text{ within structural model }X=0\\ \;Y_{w}^{*}=0+\alpha_{11}\cdot Z_{w}^{*}+\alpha_{12}\cdot V_{w}^{*}+\zeta_{w}\\ \text{ within structural model }X=1\\ (\begin{array}{c} Y_{b}^{*}\\ Z_{b}^{*}\\ V_{b}^{*} \end{array})=(\begin{array}{c} \begin{array}{c} \beta_{00} \end{array}\\ \mu_{01}\\ \mu_{02} \end{array})+(\begin{array}{c} \begin{array}{c} \beta_{01} \end{array}\\ 0\\ 0 \end{array})Z_{b}^{*}+(\begin{array}{c} \begin{array}{c} \beta_{02} \end{array}\\ 0\\ 0 \end{array})V_{b}^{*}+(\begin{array}{c} \begin{array}{c} \begin{array}{c} \zeta_{1b} \end{array} \end{array}\\ \zeta_{2b}\\ \zeta_{3b} \end{array})\\ \text{ between structural model }X=0\\ (\begin{array}{c} Y_{b}^{*}\\ Z_{b}^{*}\\ V_{b}^{*} \end{array})=(\begin{array}{c} \begin{array}{c} \beta_{10} \end{array}\\ \mu_{11}\\ \mu_{12} \end{array})+(\begin{array}{c} \begin{array}{c} \begin{array}{c} \beta_{11} \end{array} \end{array}\\ 0\\ 0 \end{array})Z_{b}^{*}+(\begin{array}{c} \begin{array}{c} \begin{array}{c} \beta_{12} \end{array} \end{array}\\ 0\\ 0 \end{array})V_{b}^{*}+(\begin{array}{c} \begin{array}{c} \begin{array}{c} \begin{array}{c} \zeta_{1b} \end{array} \end{array} \end{array}\\ \zeta_{2b}\\ \zeta_{3b} \end{array})\\ \text{ between structural model }X=1 \end{array}$$

Marsh et al. (2009) termed this model a “Manifest-Latent Model.” The computations of the *g*_1_(Z_b_^*^, V_b_^*^, Z_w_^*^, V_w_^*^) and the average treatment effect mimic the corresponding computations shown for M3 (see Equations 11, 12):

$$\begin{array}{l} g_{1}(Z_{b}^{*},V_{b}^{*},Z_{w}^{*},V_{w}^{*})=(\beta_{10}-\beta_{00})+(\beta_{11}-\beta_{01})\cdot Z_{b}^{*}\\ \;+\;(\beta_{12}-\beta_{02})\cdot V_{b}^{*}+(\alpha_{11}-\alpha_{01})\cdot Z_{w}^{*}\\ \;+\;(\alpha_{12}-\alpha_{02})\cdot V_{w}^{*}\\ \;=\gamma_{10}+\gamma_{11}\cdot Z_{b}^{*}+\gamma_{12}\cdot V_{b}^{*}+\gamma_{13}\cdot Z_{w}^{*}\\ \;+\;\gamma_{14}\cdot V_{w}^{*}\\ \;AVE=(\beta_{10}-\beta_{00})+(\beta_{11}-\beta_{01})\cdot E(Z)\\ \;+\;(\beta_{12}-\beta_{02})\cdot E(V) \end{array}$$

### 7.5. M5: MG-ML-SEM latent covariates/manifest aggregation

In our fifth MG-ML-SEM, we take one step back and again apply manifest aggregation for covariates. Unlike previous models, we include a measurement model for the latent covariate “interest in reading,” i.e., the three positively worded items *V*_1_, *V*_2_, *V*_3_ are indicators of a latent construct η. The three indicators are decomposed into their within- and between-cluster components using manifest aggregation. In the terminology of Marsh et al. (2009), M5 is called a “Latent-Manifest Model.” Particular advantages of explicitly including a latent covariate are: (1) one can appropriately account for measurement error in the covariate, (2) the option to test measurement invariance across groups and across levels, and (3) the availability of fit indices to examine model fit. Adding a latent variable in the MG-ML-SEM approach requires the specification of a within-cluster measurement model and a between-cluster measurement model for both treatment groups. We assume measurement invariance across levels (Mehta and Neale, 2005; Jak et al., 2013) and across groups (Meredith, 1993). In the model equations, we show the common within-cluster and the common between-cluster measurement models:

$$\begin{array}{l} (\begin{array}{c} \begin{array}{c} V_{1w} \end{array}\\ V_{2w}\\ V_{3w} \end{array})=(\begin{array}{c} 1\\ \lambda_{2}\\ \lambda_{3} \end{array})\eta_{w}+(\begin{array}{c} \varepsilon_{1w}\\ \varepsilon_{2w}\\ \varepsilon_{3w} \end{array})\\ \text{ within measurement model}\\ (\begin{array}{c} \begin{array}{c} \begin{array}{c} V_{1b} \end{array} \end{array}\\ V_{2b}\\ V_{3b} \end{array})=(\begin{array}{c} 0\\ \nu_{2}\\ \nu_{3} \end{array})+(\begin{array}{c} \begin{array}{c} 1 \end{array}\\ \lambda_{2}\\ \lambda_{3} \end{array})\eta_{b}+(\begin{array}{c} \begin{array}{c} \varepsilon_{1b} \end{array}\\ \varepsilon_{2b}\\ \varepsilon_{3b} \end{array})\\ \text{ between measurement model}\\ \;Y_{w}^{*}=0+\alpha_{01}\cdot Z_{w}+\alpha_{02}\cdot \eta_{w}+\zeta_{w}\\ \text{ within structural model }X=0\\ \;Y_{w}^{*}=0+\alpha_{11}\cdot Z_{w}+\alpha_{12}\cdot \eta_{w}+\zeta_{w}\\ \text{ within structural model }X=1\\ (\begin{array}{c} \begin{array}{c} Y_{b}^{*} \end{array}\\ Z_{b}\\ \eta_{b} \end{array})=(\begin{array}{c} \begin{array}{c} \beta_{00} \end{array}\\ \mu_{01}\\ \mu_{02} \end{array})+(\begin{array}{c} \begin{array}{c} \begin{array}{c} \beta_{01} \end{array} \end{array}\\ 0\\ 0 \end{array})Z_{b}+(\begin{array}{c} \begin{array}{c} \begin{array}{c} \beta_{02} \end{array} \end{array}\\ 0\\ 0 \end{array})\eta_{b}+(\begin{array}{c} \begin{array}{c} \begin{array}{c} \begin{array}{c} \zeta_{1b} \end{array} \end{array} \end{array}\\ \zeta_{2b}\\ \zeta_{3b} \end{array})\\ \text{ between structural model }X=0\\ (\begin{array}{c} \begin{array}{c} Y_{b}^{*}\\ Z_{b}\\ \eta_{b} \end{array} \end{array})=(\begin{array}{c} \begin{array}{c} \begin{array}{c} \beta_{10} \end{array}\\ \mu_{11}\\ \mu_{12} \end{array} \end{array})+(\begin{array}{c} \begin{array}{c} \begin{array}{c} \begin{array}{c} \beta_{11} \end{array} \end{array}\\ 0\\ 0 \end{array} \end{array})Z_{b}+(\begin{array}{c} \begin{array}{c} \begin{array}{c} \begin{array}{c} \beta_{12} \end{array} \end{array}\\ 0\\ 0 \end{array} \end{array})\eta_{b}+(\begin{array}{c} \begin{array}{c} \begin{array}{c} \begin{array}{c} \begin{array}{c} \zeta_{1b} \end{array} \end{array} \end{array}\\ \zeta_{2b}\\ \zeta_{3b} \end{array} \end{array})\\ \text{ between structural model }X=1 \end{array}$$

Average and conditional effects are computed as in the previous models. Note that we use the unconditional expectation of the latent covariate η to compute the average treatment effect. *E*(η) will depend on the chosen scale: In a τ-congeneric measurement model as used in this example, η is uniquely defined up to positive linear transformations. We fixed the scale of η by fixing the first measurement intercept to ν_1_ = 0 and the first loading to λ_1_ = 1 in both treatment groups^3^. Given a group-invariant measurement model, *E*(η) can be computed:

$$E(\eta )=\mu_{02}\cdot P(X=0)+\mu_{12}\cdot P(X=1).$$

The effect function and the *AVE* from M5 are then (see Equations 11, 12):

$$\begin{array}{l} g_{1}(Z_{b},\eta_{b},Z_{w},\eta_{w})=(\beta_{10}-\beta_{00})+(\beta_{11}-\beta_{01})\cdot Z_{b}\\ \;+\;(\beta_{12}-\beta_{02})\cdot \eta_{b}+(\alpha_{11}-\alpha_{01})\cdot Z_{w}\\ \;+\;(\alpha_{12}-\alpha_{02})\cdot \eta_{w}\\ \;=\gamma_{10}+\gamma_{11}\cdot Z_{b}+\gamma_{12}\cdot \eta_{b}+\gamma_{13}\cdot Z_{w}\\ \;+\;\gamma_{14}\cdot \eta_{w}\\ \;AVE=(\beta_{10}-\beta_{00})+(\beta_{11}-\beta_{01})\cdot E(Z)\\ \;+\;(\beta_{12}-\beta_{02})\cdot E(\eta ) \end{array}$$

### 7.6. M6: MG-ML-SEM latent covariates/latent aggregation

The sixth and final model is the full doubly latent MG-ML-SEM including latent covariates and latent aggregation. The only difference compared to M5 is that the aggregation of pre-treatment reading comprehension *Z* and the three indicators of interest in reading is latent as indicated by the superscript of an asterisk. M6 is specified by the following equations:

$$\begin{array}{l} (\begin{array}{c} V_{1w}^{*}\\ V_{2w}^{*}\\ V_{3w}^{*} \end{array})=(\begin{array}{c} \begin{array}{c} 1 \end{array}\\ \lambda_{2}\\ \lambda_{3} \end{array})\eta_{w}^{*}+(\begin{array}{c} \begin{array}{c} \varepsilon_{1w} \end{array}\\ \varepsilon_{2w}\\ \varepsilon_{3w} \end{array})\\ \text{ within measurement model}\\ (\begin{array}{c} \begin{array}{c} V_{1b}^{*}\\ V_{2b}^{*}\\ V_{3b}^{*} \end{array} \end{array})=(\begin{array}{c} \begin{array}{c} \begin{array}{c} 0 \end{array}\\ \nu_{2}\\ \nu_{3} \end{array} \end{array})+(\begin{array}{c} \begin{array}{c} \begin{array}{c} 1 \end{array}\\ \lambda_{2}\\ \lambda_{3} \end{array} \end{array})\eta_{b}^{*}+(\begin{array}{c} \begin{array}{c} \begin{array}{c} \varepsilon_{1b} \end{array}\\ \varepsilon_{2b}\\ \varepsilon_{3b} \end{array} \end{array})\\ \text{ between measurement model}\\ \;Y_{w}^{*}=0+\alpha_{01}\cdot Z_{w}^{*}+\alpha_{02}\cdot \eta_{w}^{*}+\zeta_{w}\\ \text{ within structural model }X=0\\ \;Y_{w}^{*}=0+\alpha_{11}\cdot Z_{w}^{*}+\alpha_{12}\cdot \eta_{w}^{*}+\zeta_{w}\\ \text{ within structural model }X=1\\ (\begin{array}{c} \begin{array}{c} Y_{b}^{*} \end{array}\\ Z_{b}^{*}\\ \eta_{b}^{*} \end{array})=(\begin{array}{c} \begin{array}{c} \begin{array}{c} \beta_{00} \end{array} \end{array}\\ \mu_{01}\\ \mu_{02} \end{array})+(\begin{array}{c} \begin{array}{c} \begin{array}{c} \begin{array}{c} \beta_{01} \end{array} \end{array} \end{array}\\ 0\\ 0 \end{array})Z_{b}^{*}+(\begin{array}{c} \begin{array}{c} \begin{array}{c} \begin{array}{c} \beta_{02} \end{array} \end{array} \end{array}\\ 0\\ 0 \end{array})\eta_{b}^{*}+(\begin{array}{c} \begin{array}{c} \begin{array}{c} \begin{array}{c} \begin{array}{c} \zeta_{1b} \end{array} \end{array} \end{array} \end{array}\\ \zeta_{2b}\\ \zeta_{3b} \end{array})\\ \text{ between structural model }X=0\\ (\begin{array}{c} \begin{array}{c} Y_{b}^{*} \end{array}\\ Z_{b}^{*}\\ \eta_{b}^{*} \end{array})=(\begin{array}{c} \begin{array}{c} \begin{array}{c} \beta_{10} \end{array} \end{array}\\ \mu_{11}\\ \mu_{12} \end{array})+(\begin{array}{c} \begin{array}{c} \begin{array}{c} \begin{array}{c} \beta_{11} \end{array} \end{array} \end{array}\\ 0\\ 0 \end{array})Z_{b}^{*}+(\begin{array}{c} \begin{array}{c} \begin{array}{c} \begin{array}{c} \beta_{12} \end{array} \end{array} \end{array}\\ 0\\ 0 \end{array})\eta_{b}^{*}+(\begin{array}{c} \begin{array}{c} \begin{array}{c} \begin{array}{c} \begin{array}{c} \zeta_{1b} \end{array} \end{array} \end{array} \end{array}\\ \zeta_{2b}\\ \zeta_{3b} \end{array})\\ \text{ between structural model }X=1 \end{array}$$

**Figure 1:**
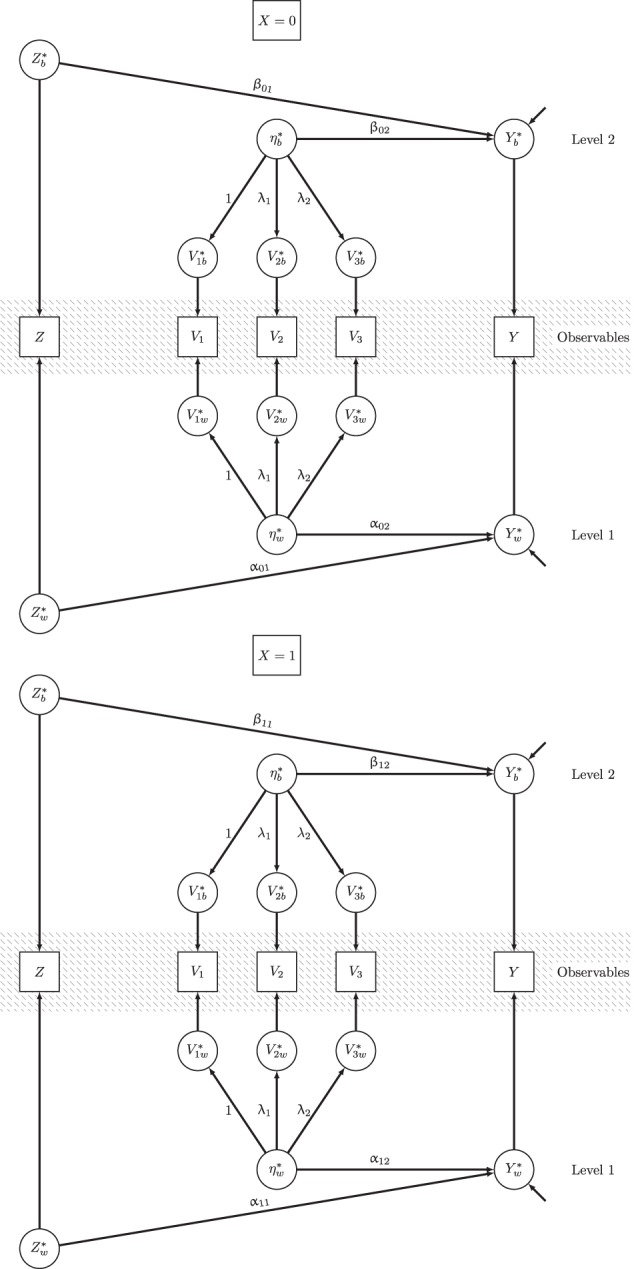
**Path diagram for the full doubly latent multigroup multilevel structural equation model**.

Figure 1 illustrates M6. The upper path diagram of Figure 1 refers to treatment group *X* = 0 and the lower path diagram refers to *X* = 1. Each of the group-specific path diagrams is divided into three parts: the gray-shaded middle part shows the observed variables that are decomposed into contextual variables (upper part L2) and unit-level residuals (lower part L1). Based on the parameters of the doubly latent model shown in the model equations above and displayed in Figure 1, we can compute the effect function and its expectation by (see Equations 11, 12):

$$\begin{array}{l} g_{1}(Z_{b}^{*},\eta_{b}^{*},Z_{w}^{*},\eta_{w}^{*})=(\beta_{10}-\beta_{00})+(\beta_{11}-\beta_{01})\cdot Z_{b}^{*}\\ \;+\;(\beta_{12}-\beta_{02})\cdot \eta_{b}^{*}+(\alpha_{11}-\alpha_{01})\cdot Z_{w}^{*}\\ \;+\;(\alpha_{12}-\alpha_{02})\cdot \eta_{w}^{*}\\ \;=\gamma_{10}+\gamma_{11}\cdot Z_{b}^{*}+\gamma_{12}\cdot \eta_{b}^{*}+\gamma_{13}\cdot Z_{w}^{*}\\ \;+\;\gamma_{14}\cdot \eta_{w}^{*}\\ \;AVE=(\beta_{10}-\beta_{00})+(\beta_{11}-\beta_{01})\cdot E(Z)\\ \;+\;(\beta_{12}-\beta_{02})\cdot E(\eta ) \end{array}$$

Mplus syntax for M6 is given in the Supplementary Material.

## 8. Results

The aim of our illustrative example is twofold: First, we demonstrate the consequences of estimating average effects with different models. Second, we show how to use the MG-ML-SEM approach to estimate conditional effects and average effects of the treated and the untreated. We begin our presentation with the descriptive statistics of the central variables in our analyses.

### 8.1. Descriptive statistics

Table 2 shows means, standard deviations, and intra-class correlation coefficients for all manifest variables used in our analyses. The left-hand columns depicts descriptive statistics for those students that stayed in elementary school until the end of 6th grade (the control group *X* = 0), whereas the right-hand columns depicts descriptive statistics for those students that made the early transition to secondary school after 4th grade (the treatment group, *X* = 1). Descriptively, the distributions of pre-treatment covariates are quite different between the two groups, which is expected in quasi-experimental designs like the ELEMENT study.

**Table 2:** **Means, standard deviations, and intra-class correlation coefficients for all manifest variables used in the analyses**.

	**Elementary school**			**Secondary school**		
**Variable**	***M***	***SD***	***ICC***	***M***	***SD***	***ICC***
*Y*	109.60	12.71	0.24	123.40	10.61	0.10
*Z*	97.33	15.12	0.21	113.84	11.27	0.09
*V*	2.98	0.78	0.04	3.37	0.58	0.04
*V*_1_	2.31	1.07	0.02	2.72	0.97	0.02
*V*_2_	3.37	0.90	0.02	3.65	0.64	0.02
*V*_3_	3.27	0.99	0.03	3.71	0.63	0.02

*Y: Reading comprehension 6th grade; *Z*: Reading comprehension 4th grade; *V*: Manifest scale interest in reading; *V*_1_, *V*_2_, *V*_3_: Three positively worded items of scale interest in reading*.

### 8.2. Comparison of models

The first aim of our illustrative example was to examine whether model choice is important for assessing the effects of early transition to secondary school on reading comprehension. To examine the differences between the models, we analyzed the data from the ELEMENT study with the six MG-ML-SEMs presented in the last section. All models were implemented using M*plus 7* (Muthén and Muthén, 2012). Table 3 shows the average treatment effect together with standard errors and effect sizes, the parameters of the conditional effect functions *g*_1_(*Z*), and group-specific contextual effects of covariates for all models. Effect sizes (*ES*) for the average effects were computed by dividing *AVE* by the standard deviation of *Y* given *X* = 0. The substantive conclusions drawn from the models are quite different, as will be discussed in detail in the following paragraphs.

**Table 3:** **Average and conditional effects obtained from six multigroup multilevel structural equation models**.

**Parameter**	**M1**		**M2**		**M3**		**M4**		**M5**		**M6**	
	**Est**.	**SE**	**Est**.	**SE**	**Est**.	**SE**	**Est**.	**SE**	**Est**.	**SE**	**Est**.	**SE**
**PARAMETERS OF CONDITIONAL EFFECT FUNCTIONS (*g*_1_(*Z*)) AND AVERAGE EFFECTS**												
γ_10_	13.59	0.76	16.43	3.36	22.17	9.77	11.67	15.82	11.99	11.28	10.14	15.75
γ_11_			−0.12	0.03	−0.21	0.12	−0.13	0.16	−0.15	0.12	−0.14	0.21
γ_12_			0.40	0.50	0.44	2.78	0.55	5.11	1.88	3.48	1.60	9.27
γ_13_			−0.12	0.03	−0.10	0.03	−0.10	0.03	−0.10	0.03	−0.09	0.03
γ_14_			0.40	0.50	0.36	0.52	0.27	0.52	0.44	0.92	0.34	0.90
*AVE*	13.59	0.76	5.23	0.63	1.76	1.00	0.14	1.38	1.33	1.12	0.15	1.41
*ES*	1.07		0.43		0.14		0.01		0.10		0.01	
**GROUP-SPECIFIC CONTEXTUAL EFFECTS OF COVARIATES**												
*CE*_*ZY;X*=0_					0.27	0.04	0.31	0.04	0.28	0.03	0.31	0.04
*CE*_*ZY;X*=1_					0.16	0.13	0.28	0.17	0.23	0.12	0.27	0.22
*CE*_*VY;X*=0_					1.09	1.09	1.46	2.26	−0.79	1.42	0.77	3.11
*CE*_*VY;X*=1_					1.17	2.66	1.73	4.70	0.64	3.20	2.03	8.74

*The effect function is given by g_1_(Z) = γ_10_ + γ_11_ Z_b_ + γ_12_ V_b_ + γ_13_ Z_w_ + γ_14_ V_w_ (in models with latent variables, V is replaced by η; in models using latent aggregation of covariates, a superscript of an asterisk is added to covariates). The values of the effect function are the* (**Z** = **z**)-*conditional effects of early transition to secondary school. The average treatment effect is AVE = E[g_1_(Z)]. ES denotes the effect size for the average effect obtained by dividing AVE by the standard deviation of Y given X = 0. Contextual effects are the differential effects of the within-cluster and between-cluster components of the covariate on the outcome variable. Contextual effects are calculated by subtracting the within-coefficient from the between-coefficient given X = x, i.e., CE_ZY;X = x_ = β_x1_ − α_x1_ and CE_VY;X = x_ = β_x2_ − α_x2_. In M1 and M2, there are no contextual effects*.

There was a strong and significant average treatment effect obtained from M1 (*AVE* = 13.59, 95% *CI* [12.10, 15.08], *ES* = 1.07). In a randomized experiment, an applied researcher might conclude that, on average, early transition to secondary school was beneficial for students' reading comprehension. However, in the present quasi-experimental setting, the observed difference in outcome means between treatment groups may be due to pre-existing differences. In fact, as shown in Table 2, those students who chose to make the early transition to secondary school already have higher values on reading comprehension in 4th grade and higher interest in reading on average. Therefore, we need to control for these pre-existing differences by conditioning on relevant covariates.

In M2, we controlled for the manifest covariates “reading comprehension in 4th grade” *Z* and “interest in reading” *V* without distinguishing within and between components of these covariates. The average treatment effect obtained from M2 (*AVE* = 5.23, 95% *CI* [3.90, 6.47], *ES* = 0.43) was considerably lower compared to the *AVE* from M1 but is still significant. If there were no contextual effects and no other covariates introducing bias, one would conclude that early transition to secondary school positively affects students' reading comprehension on average.

M3 is the first model in our sequence of models that separated the within-cluster and between-cluster components of covariates and estimates contextual effects. In our illustrative example, there was a significant contextual effect for reading comprehension at 4th grade for those students that stayed in primary schools (*CE*_*ZY;X* = 0_ = 0.27, 95% *CI* [0.19, 0.35]), but no significant contextual effect of reading comprehension at 4th grade for students that moved to secondary schools and no contextual effects for interest in reading in both groups (see Table 3). Substantively, the contextual effect showed that students with equal initial achievement levels and equal interest in reading (i.e., given *Z = z* and *V = v*) profited from being schooled together with high achieving students (i.e., high values on *Z*_*b*_ and *V*_*b*_) in primary school, but not if they made an early transition to secondary school. In M3, where we took account of contextual covariates in the computation of the average treatment effect, the *AVE* was no longer significant (*AVE* = 1.76, 95% *CI* [−0.20, 3.72], *ES* = 0.14).

All subsequent models (M4–M6) revealed a similar pattern of results: The *AVE* is not significant and there was a contextual effect of initial reading comprehension for students that stayed in primary school. The estimates from models M3–M6 ranged from *AVE* = 1.76 (95% *CI* [−0.20, 3.72], *ES* = 0.14; M3) to 0.14 (95% *CI* [−2.57, 2.85], *ES* = 0.01; M4). Descriptively, the models using latent aggregation of covariates (M4 and M6) show the lowest *AVE*. When the effect estimates were standardized using the standard deviation of the outcome in primary school, the results were very similar (see row *ES* in Table 3). With regard to standard errors, we find that the models including latent aggregation (M4 and M6) had the highest standard errors, followed by the models using manifest aggregation and contextual covariates (M3 and M5), and the model ignoring contextual covariates (M2) had the lowest standard error. M6 had the highest standard error, because it accounts for uncertainty in the aggregation of covariates and also includes latent covariates.

In summary, the average total effects obtained from models M3–M6 were pretty similar, but they were substantially different from the apparent effects obtained from M1 and M2. The comparison of models clearly shows that it is necessary to control for contextual covariates, if there are contextual effects.

### 8.3. Doubly-latent model M6

In order to further illustrate the advantages of the MG-ML-SEM approach, we present a more detailed analysis of conditional and average effects based on the doubly-latent model M6. Although there were only minor differences between M3 and M6 in our illustrative example, we generally prefer M6, because it overcomes all of the limitations of conventional ANCOVA mentioned in the introduction. We note, however, that there might be situations in which partial correction models may be more appropriate for substantive or statistical reasons (Lüdtke et al., 2008, 2011; Marsh et al., 2012).

Detailed results for all parameters of the doubly-latent model M6 and model fit information are given in Table 4. The effect function *g*_1_(*Z*), the average treatment effect *AVE*, contextual effects, and all conditional effects considered in subsequent paragraphs, were based on these parameters. Figure 1 shows a path diagram of the full model. Next, we demonstrate how the MG-ML-SEM approach could be used to answer substantive research questions using our preferred model (M6) as case example. In particular, we show how the MG-ML-SEM approach can be used to examine average effects as well as conditional treatment effects given certain values of covariates.

**Table 4:** **Results obtained from the full doubly latent multigroup multilevel structural equation model**.

	**Model parameters**			
	***X* = 0**		***X* = 1**	
	**Within**	**Between**	**Within**	**Between**
**MEASUREMENT MODEL**				
ν_1_	–	0.00^*^	–	0.00^*^
ν_2_	–	0.81 (0.10)	–	0.81 (0.10)
ν_3_	–	0.09 (0.15)	–	0.09 (0.15)
λ_1_	1.00^*^	1.00^*^	1.00^*^	1.00^*^
λ_2_	1.07 (0.04)	1.07 (0.04)	1.07 (0.04)	1.07 (0.04)
λ_3_	1.36 (0.06)	1.36 (0.06)	1.36 (0.06)	1.36 (0.06)
*Var*(ε_1_)	0.86	0.00^*^	0.76	0.00^*^
*Var*(ε_2_)	0.44	0.00^*^	0.22	0.00^*^
*Var*(ε_3_)	0.37	0.00^*^	0.12	0.00^*^
**STRUCTURAL MODEL**				
α_*x*1_	0.52 (0.01)	–	0.42 (0.02)	–
α_*x*2_	2.31 (0.41)	–	2.65 (0.79)	–
β_*x*0_	–	21.62 (6.47)	–	31.76 (14.34)
β_*x*1_	–	0.83 (0.04)	–	0.70 (0.21)
β_*x*2_	–	3.08 (2.98)	–	4.68 (8.75)
μ_*x*1_	–	97.40 (0.65)	–	113.64 (0.52)
μ_*x*2_	–	2.34 (0.02)	–	2.67 (0.03)
*Var*(ζ)	69.54	2.58	77.44	5.11
**MODEL FIT**				
χ^2^ (30) = 175.60				
RMSEA = 0.04				
SRMR (within) = 0.04				
SRMR (between) = 0.10				

*Parameters with a superscript of an asterisk are fixed parameters. ν_1_ to ν_3_ are invariant intercepts; λ_1_ to λ_3_ are invariant loadings; Var(ε_1_) − Var(ε_3_) denote variances of measurement error variables; α_x1_-α_x2_ are regression coefficients at the within-cluster level; β_x1_-β_x2_ are regression coefficients at the between-cluster level; μ_x1_ and μ_x2_ are group-specific means of covariates; Var(ζ) denotes the variance of the regression residuals*.

In our illustrative example, the average effect is not the only interesting quantity from a substantive point of view. When evaluating an intervention it is not only of interest *if* there is an average effect, but also *for whom* the intervention is beneficial or even harmful. Early transition to secondary school might not have a significant average effect, but could still affect specific students, e.g., students with high or low values of pre-treatment covariates. For example, high achieving students might particularly benefit from an early transition to secondary schooling, whereas the early transition might negatively impact students with low initial achievement. The MG-ML-SEM approach can be used to study such conditional effects. In addition, it is also possible to study average treatment effects for certain sub-populations. From a policy perspective, it might be interesting to study the effect of the early transition on the group of students actually choosing early transition to secondary school (this effect has also be termed the “effect on the treated”). Or one might be interested in whether early transition to secondary school would have a beneficial effect for those who did not take this opportunity (“effect on the untreated”).

Prior to estimating M6 for computing average and conditional effects, we tested for measurement invariance of “interest in reading” by comparing a model with invariance across groups and across levels (χ^2^ = 81.57, *df* = 14, *p* < 0.001; BIC = 28192.23; RMSEA = 0.05; CFI = 0.97; TLI = 0.98) with a model where invariance was not assumed (χ^2^ = 1.69, *df* = 6, *p* = 0.95; BIC = 28198.01; RMSEA = 0.00; CFI = 1.00; TLI = 1.00)^4^. While the χ^2^-test of model fit was significant for the model with invariance, the other fit measures indicated a good fitting model, and the BIC was lower for the model with invariance. We concluded that the more parsimonious model with measurement invariance is adequate.

Based on M6, we first tested the null hypothesis that all conditional effects were equal using a Wald test, i.e., *H*_0_: γ_11_ = γ_12_ = γ_13_ = γ_14_ = 0, where γ_11_ to γ_14_ are regression coefficients in the effect function (cf. Table 3). Average effects on the treated and the untreated will differ from the average treatment effect only if the conditional effects are not constant. It is one of the particular strengths of the MG-ML-SEM approach that it allows tests of the treatment-covariate interactions and does not presume that conditional treatment effects are constant. Based on the Wald test, we rejected the null hypothesis (χ^2^ = 14.25, *df* = 4, *p* = 0.0065) of constant treatment effects.

Next, we considered conditional treatment effects, such as the conditional effect of early transition to secondary school on the treated (CTET). The CTET is the conditional expectation of the effect function given *X* = 1, i.e., CTET = *E*[*g*_1_(Z^*^_b_, Z^*^_w_,η^*^_b_,η^*^_w_) | *X* = 1]. It can be computed based on the parameters of the model (see Table 4):

$$\begin{array}{l} E[g_{1}(Z_{b}^{*},Z_{w}^{*},\eta_{b}^{*},\eta_{w}^{*})\;|\;X=1]=\gamma_{10}+\gamma_{11}\cdot E(Z_{b}^{*}\;|\;X=1)\\ \;+\;\gamma_{12}\cdot E(\eta_{b}^{*}\;|\;X=1)\\ \;=(\beta_{10}-\beta_{00})+(\beta_{11}-\beta_{01})\cdot \mu_{11}\\ \;+\;(\beta_{12}-\beta_{02})\cdot \mu_{12} \end{array}$$

using the fact that the conditional expectations of within-cluster components Z^*^_w_ and η^*^_w_ given *X* = *x* is zero^5^. In our illustrative example, the CTET was not significant (*CTET* = −0.92, 95% *CI* [−3.00, 1.16]). This implies that the early transition to secondary schooling was not beneficial for the group of students that actually underwent this transition. If this effect was causally interpretable (which would require controlling a larger set of covariates), it would imply that there were no beneficial effects of the supposedly enriched learning environment of the secondary school for the group of students that went there. These students would have obtained similar reading outcomes in the 6th grade after attending primary schools.

Similarly, the conditional treatment effect on the untreated (CTEUT) is the effect that early transition to secondary school would have on students who stay in elementary school until the end of sixth grade. The CTEUT can be computed as the conditional expectation of the effect function given *X* = 0:

$$\begin{array}{l} E[g_{1}(Z_{b}^{*},Z_{w}^{*},\eta_{b}^{*},\eta_{w}^{*})\;|\;X=0]=\gamma_{10}+\gamma_{11}\cdot E(Z_{b}^{*}\;|\;X=0)\\ \;+\;\gamma_{12}\cdot E(\eta_{b}^{*}\;|\;X=0)\\ \;=(\beta_{10}-\beta_{00})+(\beta_{11}-\beta_{01})\cdot \mu_{01}\\ \;+\;(\beta_{12}-\beta_{02})\cdot \mu_{02}. \end{array}$$

In our analyses, the CTEUT is also not significant (*CTEUT* = 0.74, 95% *CI* [−3.28, 4.76]). Again, under the assumption that all relevant covariates had been controlled, this would imply that students who stayed in primary school longer would not have profited from an earlier transition to secondary schooling with respect to their reading proficiency.

Furthermore, we can consider conditional effects for specific values of covariates. For example, we might be interested in the conditional effect for an average elementary school student (*Z* = 97.40, η = 2.34) in an average secondary school class (Z^*^_b_ = 113.64, η^*^_b_ = 2.67), i.e., with Z^*^_w_ = *Z* − Z^*^_b_ = −16.24 and η^*^_w_ = η − η^*^_b_ = −0.33:

$$\begin{array}{l} g_{1}(z)=\gamma_{10}+\gamma_{11}\cdot 113.64+\gamma_{12}\cdot 2.67+\gamma_{13}\cdot (-16.24)\\ \;+\;\gamma_{14}\cdot (-0.33), \end{array}$$

which yields *CTE* = 0.49 (95% *CI* [−1.96, 2.94]). Or we might be interested in the conditional effect for an average secondary school student (*Z* = 113.64, η = 2.67) in an average elementary school class (Z^*^_b_ = 97.40, η^*^_b_ = 2.34), i.e., with Z^*^_w_ = *Z* − Z^*^_b_ = 16.24 and η^*^_w_ = η − η^*^_b_ = 0.33:

$$g_{1}(z)=\gamma_{10}+\gamma_{11}\cdot 97.40+\gamma_{12}\cdot 2.34+\gamma_{13}\cdot 16.24+\gamma_{14}\cdot 0.33,$$

which yields *CTE* = −0.66 (95% *CI* [−4.95, 3.63]). Both of these conditional effects are not significant.

## 9. Discussion

In this paper, we presented the MG-ML-SEM implementation of generalized ML-ANCOVA for the analysis of quasi-experimental multilevel designs with non-randomized assignment at the cluster-level. We demonstrated the flexibility of this approach and its potential for the analyses of average and conditional treatment effects using data from a German educational study focusing on early transitions to secondary schooling. The MG-ML-SEM approach overcomes the limitations of conventional multilevel ANCOVA by (1) accounting for measurement error, (2) systematically including contextual covariates with appropriate control for sampling error, (3) naturally including treatment-covariate interactions as a default and (4) treating predictors as stochastic rather than fixed. We now discuss some limitations of our example and focus on the conditions for interpreting average and conditional effect estimates obtained from generalized multilevel ANCOVA as causal effects.

### 9.1. Causal inference

Causal effects are crucial in theories in the social sciences. Almost all theories include statements that can be formalized as statements about causal effects of a variable *X* on a variable *Y*. Many researchers have the idea that a causal treatment effect is what is estimated by a mean difference (between treatment and control) in a randomized experiment. Although it turns out that this idea is not wrong, it does not help much in situations in which it is not possible or desirable to randomly assign persons/clusters to treatments. How and under which conditions can we estimate a causal effect? The general conclusion is that specific techniques of causal modeling are indispensable, whenever we are beyond total effects in a randomized experiment.

Approaches to causality include Rubin's causal model (Rubin, 1974), Pearl's graphical approach (Pearl, 2009), the stochastic theory of causal effects (Steyer et al., 2014), or Dawid's approach (Dawid, 2011). These theories of causality provide definitions of causal effects and point out the assumptions required to estimate average and conditional causal effects from empirically estimable conditional expectations in experimental and quasi-experimental designs.

A requirement of a causal analysis is a temporal structure, i.e., the focused cause has to be prior to the outcome, and the covariates have to be prior or simultaneous to the cause, so that the covariates can not be affected by the cause. This distinguishes covariates from potential mediators. The temporal structure of random variables can either be described by a directed acyclic graph (DAG; Pearl, 2009) or using a filtration (Steyer et al., 2014). In the ELEMENT study, the longitudinal aspect of the study makes such a time order plausible: The covariates “interest in reading” and “pre-test reading comprehension” are measured at 4th grade, prior to the focused cause “early transition to secondary school,” and the outcome “post-test reading comprehension” is measured at 6th grade which is posterior to early transition.

One strategy for identifying causal effects is to control for all confounders. In informal terms, a confounder is a covariate that will bias the effects of the focused cause on the outcome, if we do not control for it appropriately (see Steyer et al., 2014, for a definition of unbiasedness). Applied researchers are encouraged to test whether a covariate is a confounder or not: In Pearl's theory, the (conditional) independence statements implied by the DAG can be tested. Based on a correctly specified DAG, the researcher can then read off the covariates that need to be controlled in a causal analysis using the backdoor criterion (Pearl, 1993). In the stochastic theory of causal effects, there are several testable causality conditions that imply unbiasedness (Steyer et al., 2000, 2014). For example, the independent cause condition is defined as conditional independence of the cause and all covariates given the selected covariates in the model (see CC1Z in Steyer et al., 2014). The regressively independent outcome condition (see CC2Z in Steyer et al., 2014) is defined as conditional regressive independence of the outcome variable from all covariates given the cause and the selected covariates in the model. Both of these causality conditions imply conditional unbiasedness and are testable in the sense that the corresponding conditional independence statements can be falsified in empirical studies.

Once we have identified all relevant confounders, i.e., once we have selected the covariates such that one of the causality conditions holds, we have to control for these covariates in order to estimate causal effects and not just mere associations. Based on the theories of causality mentioned above and work by others (e.g., Rosenbaum and Rubin, 1983; Robins, 1999; Shadish et al., 2002), several techniques have been developed to control for potential confounders, e.g., ANCOVA adjustment, propensity scores, weighting techniques, matching, subclassification, marginal structural models and many more (see Schafer and Kang, 2008, for an overview). In this article, we suggested an approach to analyze data from the ELEMENT study that extends ANCOVA techniques in several ways. Some of the other techniques could also be applied for analyzing data in quasi-experimental multilevel designs (see Baumert et al., 2009; Becker et al., 2014 for applications of propensity score matching to the ELEMENT data).

As mentioned previously, the primary goal of our paper was to illustrate the MG-ML-SEM approach as a means to estimate average and conditional (total) effects in educational research. The effects obtained from models M1–M6 presented in this article can only be causally interpreted if the corresponding regressions are unbiased. For example, the average effect in M1 requires unbiasedness of the conditional expectations *E*(*Y* | *X*=*x*), which is very unlikely to hold in the ELEMENT study. It would only be plausible in a randomized experiment. M2 requires unbiasedness of the group-specific regressions *E*^*X* = *x*^(*Y* | *Z*, *V*), and M3 requires unbiasedness of the group-specific regressions *E*^*X* = *x*^(*Y* | *Z_b_*, *V_b_*, *Z_w_*, *V_w_*). Similar arguments apply to the other three models presented in this paper. A careful causal analysis would require including more potential confounders and testing the causality conditions.

### 9.2. Limitations and directions for further research

As we only compared our models on an example dataset, there was no way to pick the correct model from the set of models presented. Obviously M6, the full doubly-latent model, controls for shortcomings of conventional multilevel ANCOVA, but other models could also be defended on substantive and statistical grounds. For example, M5 that does not use latent aggregation could also be appropriate as the data in the ELEMENT study has been obtained from complete samples of the students within classrooms in Grade 4. In this case, there is only a small proportion of sampling error in the aggregation of covariates to the classroom level (Lüdtke et al., 2008; Marsh et al., 2012), e.g., due to students missing the assessment date. Similarly, M4 and M5 might be more efficient in estimating the average treatment effect as the full doubly-latent model M6 has been shown to yield very variable estimates (Lüdtke et al., 2011). However, as illustrated in the empirical examples, controlling for sampling error (and measurement error) only had minor effects on the estimated effects. It was the separation into within- and between-components of the covariates that mattered most. This separation, in turn, was justified by the presence of contextual effects of achievement in the control group. In applications, bigger differences between the models are expected when sampling and measurement error increase or when contextual effects are more pronounced. In these cases, the bias of the contextual effect will be relatively larger in M3 compared to the full doubly-latent model (Lüdtke et al., 2011).

Further research on the MG-ML-SEM implementation of the generalized ML-ANCOVA should study more carefully the required sample size at both the within- and the between-level. Simulation studies suggest that there are circumstances where a sample size of at least 10 students within a class are required for reliably estimating contextual and average effects with M4 (Lüdtke et al., 2008; Nagengast, 2009) and large sample sizes at the class-level might be required for the doubly-latent model to be sufficiently accurate (Lüdtke et al., 2011). Further simulation work is needed to extend these findings to the MG-ML-SEM case and clarify what sample sizes are required for reliable inferences about average and conditional effects in this framework.

Further developments of the MG-ML-SEM framework will likely yield additional options for the application and development of generalized multilevel ANCOVA. In particular, accounting for cross-classified multilevel structures in multilevel structural equation models would be a major step forward. By cross-classified structure, we refer to a multilevel structure, which is not strictly hierarchical, but can be more complex (for details and two examples of a cross-classified structure, see Raudenbush, 1993). Properly accounting for the cross-classified structure by considering different clustering structures for the covariates and the outcomes would be particularly useful for studies such as ELEMENT were students move from one school type to another. In addition, the further development of multilevel structural equation models with latent interactions (e.g., Leite and Zuo, 2011; Nagengast et al., 2013; Schermelleh-Engel et al., 2014) could allow to include covariate-covariate interactions and other non-linear effects of latent covariates in the effect functions. Similarly, the development of three-level multilevel structural equation models will yield further opportunities for extending the models presented and allow researchers to address the complexities of multilevel quasi-experimental designs more comprehensively.

In this article, we provided applied researchers with a comprehensive toolbox to analyze average and conditional effects in non-randomized multilevel designs. We hope that this presentation encourages researchers to apply these advanced techniques to address issues of measurement error, sampling error, contextual effects, treatment-covariate interactions and ultimately causal effects in the analyses of multilevel quasi-experimental designs.

### Conflict of interest statement

The authors declare that the research was conducted in the absence of any commercial or financial relationships that could be construed as a potential conflict of interest.

## Footnotes

^1^For the presentation of the MG-ML-SEM and generalized ML-ANCOVA, we use notation for the latent aggregation approach. The corresponding equations for the manifest aggregation approach are identical except for the lack of the asterisk.

^2^True cluster-level covariates like school-funding do not have a within-cluster component.

^3^In principle, other ways of fixing the scale of η, such as fixing the latent mean and variance, are possible as well. However, we recommend this approach in the context of multigroup multilevel SEM for analyzing average and conditional effects. Using the latent variance for setting the scale would complicate specification, because the variance needs to be specified appropriately in the multigroup-multilevel setting, which requires constraints across groups and across levels. Also, we do not want to fix the mean of latent variables in each of the groups, because this would lead to biased estimates of treatment effects.

^4^Note that the fit indices differ from the ones reported in Table 4 because to test measurement invariance, we just specified the measurement model for reading interest and omitted the single-item reading comprehension measures.

^5^*E*(*Z*^*^_*w*_ | *X* = *x*) = *E*(η^*^_*w*_ | *X* = *x*) = 0, because *Z*^*^_*w*_ and η^*^_*w*_ are defined as residuals with respect to the regressor *C* (see section about contextual effects and sampling error). Therefore, *E*[*Z*^*^_*w*_ | *f*(*C*)] = *E*[η^*^_*w*_ | *f*(*C*)] = 0 for any function *f*(*C*) of *C* and the treatment variable *X* is such a function of *C* in designs with treatment application at the cluster-level considered in this paper.
