# Supplementary material for: Analyzing average and conditional effects with multigroup multilevel structural equation models
Source: Front Psychol. 2014 Apr 23;5:304. doi: 10.3389/fpsyg.2014.00304 (PMC4006036; doi:10.3389/fpsyg.2014.00304)
Supplement: Supplementary file 1 [file Presentation1.PDF]

Appendix

M*plus* Syntax

```
MODEL:
    %Within%
    zw by Asf0902@1
          Asf0903* (a)
          Asf0905* (b);

    CdlZ on AdlZ zw;
    AdlZ with zw;

    %Between%
    zb by Asf0902@1
          Asf0903* (a)
          Asf0905* (b);

    [zb*];
    [Asf0902@0 Asf0903* Asf0905*];

    CdlZ on AdlZ zb;
    AdlZ with zb;
    Asf0902@0; Asf0903@0; Asf0905@0;

MODEL 0:
    %Within%
    CdlZ on AdlZ (slpwz10)
            zw (slpwz20);
    AdlZ with zw;

    %Between%
    [AdlZ*] (mz10);
    [zb*] (mz20);
    [CdlZ*] (inty0);

    CdlZ on AdlZ (slpz10)
            zb (slpz20);
    AdlZ with zb;
    CdlZ (var0);

MODEL 1:
    %Within%
    CdlZ on AdlZ (slpwz11)
            zw (slpwz21);
    AdlZ with zw;

    %Between%
    [AdlZ*] (mz11);
```

```
    [zb*] (mz21);
    [CdlZ*] (inty1);

    CdlZ on AdlZ (slpz11)
            zb (slpz21);
    AdlZ with zb;
    CdlZ (var1);

MODEL CONSTRAINT:
    NEW(mz1 mz2 size0 size1);
    NEW(ga10 ga11 ga12 ga13 ga14 ace);
    NEW(contz1g1 contz1g2 contz2g1 contz2g2);
    NEW(vardiff);

    vardiff = var1-var0;
    size0 = 3169/4926;
    size1 = 1757/4926;
    mz1 = mz10*size0 + mz11*size1;
    mz2 = mz20*size0 + mz21*size1;

    ga10 = inty1 - inty0;
    ga11 = slpz11 - slpz10;
    ga12 = slpz21 - slpz20;
    ga13 = slpwz11 - slpwz10;
    ga14 = slpwz21 - slpwz20;
    ace = ga10 + ga11*mz1 + ga12*mz2;
    contz1g1 = slpz10 - slpwz10;
    contz1g2 = slpz11 - slpwz11;
    contz2g1 = slpz20 - slpwz20;
    contz2g2 = slpz21 - slpwz21;
```
